# Descriptive Analysis of Adverse Events Reported for New Multiple Myeloma Medications Using FDA Adverse Event Reporting System (FAERS) Databases from 2015 to 2022

**DOI:** 10.3390/ph17070815

**Published:** 2024-06-21

**Authors:** Marwan A. Alrasheed, Khalid A. Alamer, Mashael Albishi, Abdulrahman A. Alsuhibani, Omar A. Almohammed, Abdulrahman Alwhaibi, Abdullah N. Almajed, Jeff J. Guo

**Affiliations:** 1 Department of Clinical Pharmacy, College of Pharmacy, King Saud University, P.O. Box 2454, Riyadh 11451, Saudi Arabia; mashael.faiez@gmail.com (M.A.); oalmohammed@ksu.edu.sa (O.A.A.); aalwhaibi@ksu.edu.sa (A.A.); 2 Pharmacy Practice Department, College of Clinical Pharmacy, Imam Abdulrahman Bin Faisal University, Dammam 34221, Saudi Arabia; kaalamer@iau.edu.sa; 3 Department of Pharmacy Practice, College of Pharmacy, Qassim University, Buraidah 51452, Saudi Arabia; aa.alsuhibani@qu.edu.sa; 4 Pharmaceutical Care Division, King Faisal Specialist Hospital and Research Centre, MBC 11, P.O. Box 3354, Riyadh 11211, Saudi Arabia; abdullah.n.almajid@gmail.com; 5 James L. Winkle College of Pharmacy, University of Cincinnati Academic Health Center, Cincinnati, OH 45267, USA; guoje@ucmail.uc.edu

**Keywords:** cancer, multiple myeloma, pharmacovigilance

## Abstract

Background: New multiple myeloma (MM) medications have revolutionized the treatment landscape, but they are also associated with a range of adverse events (AEs). This study aims to provide a comprehensive overview of AEs reported for four new MM medications: daratumumab, ixazomib, elotuzumab, and panobinostat. Methods: This study uses a descriptive retrospective approach to analyze the FDA Adverse Event Reporting System (FAERS) from 2015 to 2022. It includes variables like medication names, report details, patient demographics, adverse events, and reporter types. The initial dataset consists of over 3700 adverse events, which are categorized into 21 groups for clarity and comparison. Results: The FAERS database revealed 367,756 adverse events (AEs) associated with novel multiple myeloma drugs from 2015–2022. Ixazomib had the highest number of reported AEs with 206,243 reports, followed by daratumumab with 98,872 reports, then elotuzumab with 26,193 AEs. Ixazomib’s AE reports increased dramatically over the study period, rising approximately 51-fold from 1183 in 2015 to 60,835 in 2022. Of the medications studied, ixazomib also recorded the highest number of deaths (24,206), followed by daratumumab (11,624), panobinostat (7227), and elotuzumab (3349). The majority of AEs occurred in patients aged 55–64 and 65–74 years. Conclusions: Ixazomib, a new MM medication, had the highest number of AEs reported. Also, it has the highest rate of reported deaths compared to other new MM medications. Clinicians should be aware of the potential AEs associated with this medication and further research is needed to understand the reasons for the high number of AEs and to develop mitigation strategies. More attention should also be paid to the safety of new multiple myeloma medications in younger patients.

## 1. Background

Multiple myeloma (MM) is a hematologic malignancy characterized by the uncontrolled growth of malignant plasma cells within the bone marrow [1]. It is characterized by the clonal proliferation of malignant plasma cells in the bone marrow and disrupts normal hematopoiesis, resulting in anemia, bone deterioration, heightened susceptibility to infections, and potential renal complications. Its etiology is multifaceted, likely stemming from a blend of genetic and environmental factors. Although it remains incurable, ongoing advancements in treatment modalities promise more efficacious disease control and enhanced patient prognoses [2,3,4]. MM is the second most common blood cancer in the United States, with an estimated 35,730 new cases and 12,590 deaths expected in 2023 [5]. This disease carries a significant mortality burden, with a relatively low five-year survival rate for multiple myeloma (MM) [6]. Additionally, the efficacy of traditional medications has been undermined by their adverse effects [7]. However, novel therapeutic agents, including proteasome inhibitors, immunomodulatory drugs, and monoclonal antibodies, have emerged recently and substantially enhanced treatment. Examples of these novel agents include daratumumab, ixazomib, elotuzumab, which received US Food and Drug Administration (FDA) approval for the treatment of MM in late 2015 [8,9,10,11,12,13,14,15]. While these newer agents have demonstrated their effectiveness, it is essential to recognize that they can also give rise to significant adverse events (AEs) [15,16,17,18].

Daratumumab (Darzalex) is a monoclonal antibody medication used to treat adults with MM. It targets CD38, a transmembrane glycoprotein that is highly expressed on multiple myeloma cells and other immune cells. Daratumumab has been shown to be highly effective in the treatment of multiple myeloma, both as a first-line therapy and in relapsed/refractory settings. It is also being investigated for the treatment of other types of cancer, such as light chain amyloidosis [8,9,19,20,21,22]

Ixazomib (Ninlaro) is an orally administered proteasome inhibitor, a multiprotein complex that is essential for cellular protein turnover. It prevents the assembly of the proteasome complex and inhibits MM cell function. This leads to the accumulation of abnormal proteins in the cell, which ultimately leads to cell death. Ixazomib is indicated for relapsed/refractory MM patients who have received at least one prior therapy [17,23,24,25,26].

Elotuzumab (Empliciti) is a monoclonal antibody that targets signaling lymphocytic activation molecule F7 (SLAMF7), a protein that is highly expressed on the surface of myeloma cells and natural killer cells. It is approved for the treatment of relapsed/refractory MM and is now considered a part of the standard of care in this setting. Elotuzumab has demonstrated significant clinical efficacy, with improved progression-free survival and overall survival [27,28,29].

Panobinostat (Farydak) is an oral pan-histone deacetylase (HDAC) inhibitor that is used to treat relapsed and refractory MM in adults [30,31,32]. In December 2021, the manufacturer of panobinostat announced that it was withdrawing the indication for panobinostat in the treatment of multiple myeloma in the United States. This decision was made after discussions with the FDA, which concluded that the clinical benefit of panobinostat had not been confirmed under the specific constraints of the accelerated approval process. Panobinostat is still available to patients in other countries, and it is still being investigated for the treatment of other types of cancer [33,34,35]. Understanding its adverse events provides valuable insights into HDAC inhibitors’ safety profiles more broadly. Moreover, patients who were treated with panobinostat during its availability might still experience long-term adverse events. Research into AEs associated with novel MM therapies remains a critical and rapidly evolving field. These efficacious agents introduce distinct AE profiles, prompting focused investigation into cardiotoxicity with certain proteasome inhibitors [36], immunosuppression-related infection risk [37], peripheral neuropathy management [38], and the potential for second primary malignancies [39]. Alongside the development of therapies with improved tolerability, current research prioritizes risk stratification and the optimization of supportive care to mitigate AEs. These multifaceted efforts are essential for enhancing treatment outcomes, improving patient quality of life, and advancing the development of safer, more effective multiple myeloma therapies.

This study is relevant and necessary, as it offers a descriptive analysis of AEs reported for new MM medications using the FAERS databases from 2015 to 2022. By utilizing FAERS data, the study contributes to ongoing medication safety surveillance, providing insights into the temporal trends and frequency of reported adverse events associated with these medications. The findings inform clinical practice by guiding healthcare professionals in patient counseling, monitoring strategies, and adverse event management. Additionally, the study adds to scientific knowledge by enhancing understanding of medication safety in multiple myeloma treatment, ultimately supporting patient safety and optimizing clinical decision-making. The FDA Adverse Event Reporting System (FAERS) serves as a national repository, collecting reports of AEs submitted to the FDA. This database compiled adverse event reports from various sources, including healthcare professionals, patients, and drug manufacturers, which provide valuable information about adverse reactions, medication errors, and product quality issues. FAERS plays a crucial role in post-market drug surveillance, helping ensure that drugs and therapeutic biologic products on the market are safe and effective for patients. It serves as an important component of pharmacovigilance efforts to monitor and improve drug safety [40].

This study aims to provide a comprehensive descriptive analysis of AEs reported in connection with these new multiple myeloma medications, using FAERS data from 2015 to 2022. The primary objectives of this analysis are as follows: first, to outline the prevalence of AEs reporting associated with the use of the new MM medications daratumumab, ixazomib, elotuzumab, and Panobinostat; second, to identify any observable trends in AE reporting over time and to assess the potential risks of these medications in the context of multiple myeloma therapy; third, to identify the outcomes associated with MM medication AEs; finally, to identify the type of reporters and their link to AEs and outcomes of MM medications. The outcomes of this study will play a pivotal role in guiding the safe and effective utilization of these new medications for the benefit of MM patients.

## 2. Results

**Figure 1:**
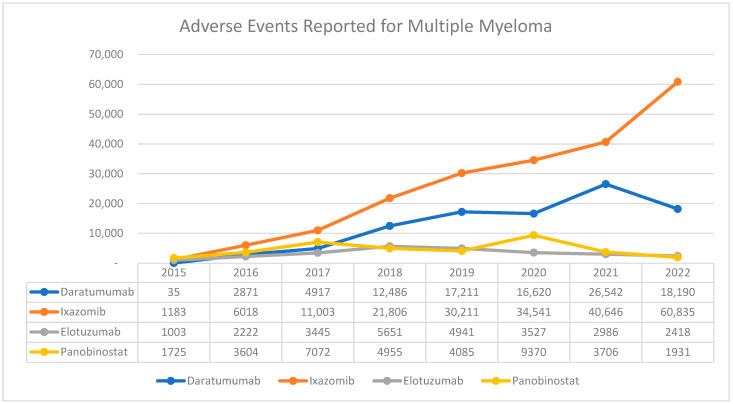
AE trends for MM medication reported to FAERS from 2015 to 2022.

From 2015 to 2022, a total of 367,756 adverse events (AEs) were reported for the new multiple myeloma medications in the FAERS databases (Figure 1). Among those, ixazomib had the highest number, with a total of 206,243 reports, followed by daratumumab, with 98,872 reports (Table 1). Elotuzumab recorded the lowest number, preceded by panobinostat with 26,193 and 36,448 reports, respectively (Table 1). It is worth noting that AEs associated with ixazomib have increased dramatically by approximately 51-fold from 1183 in 2015 to 60,835 AEs in 2022. When total AEs with all medications stratified by gender, there was a relatively equal distribution of reports between males and females, 53% vs. 47%, respectively. Although close proportions were found when conducted on each medication, elotuzumab had more reports from males compared to females (61% vs. 31%, respectively). Most of the AEs were observed in two specific age groups: 55–64 and 65–74 years (30,069, and 61,711, respectively) (Table 1). Interestingly, the occurrence of AEs among patients under the age of 18 was the highest with daratumumab (2043 reports) and ixazomib (1478 reports). Similarly, an unusual finding within our data was the number of AEs report for panobinostat 1824, specifically from individuals between the ages of 18 and 24. Table 1 provides a detailed breakdown of the adverse event reports stratified by gender and age for each medication.

### 2.1. Daratumumab

**Figure 2:**
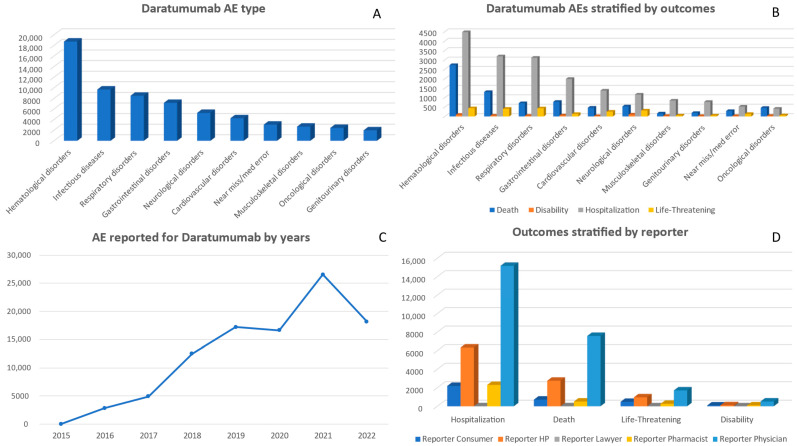
Panel of figures for daratumumab AE type, AEs stratified by outcomes, AEs by years, and outcomes stratified by type of reporter. (**A**) shows the most 10 AEs that have been reported for daratumumab. (**B**) shows daratumumab’s AEs stratified by its outcomes. (**C**) shows the trend line for daratumumab AEs from 2015 to 2022. (**D**) shows daratumumab’s AEs outcomes stratified by type of reporter.

The number of AEs linked to daratumumab revealed a noticeable rise from 2015 to 2022. The reports started with only 35 cases in 2015 and reached their peak in 2021, with a total of 26,542 reported AEs. However, there was a slight drop in these incidents in 2022, with a total number of 18,190 reports (Figure 2). Between 2015 and 2022, there were noticeable trends in the distribution of reported AEs related to daratumumab among different age groups. The highest number of reports each year was consistently observed in the age group of 65–74, reaching its peak in 2021 with a total of 9096 cases. Conversely, the age groups of 18–24 and 25–34 consistently showed the lowest number of reports, with several years recording minimal or no reports at all (Supplementary File). Among these, the most frequently reported adverse events were labeled ‘Miscellaneous’ (25,265 reports), followed by ‘Hematological disorders’ (18,733 reports), ‘Infectious diseases’ (9685 reports), ‘Respiratory disorders’ (8515 reports), and ‘Gastrointestinal disorders’ (7163 reports) (Figure 2 and Supplementary File).

The AEs associated with daratumumab have resulted in various outcomes over time. The overall deaths reported for daratumumab was 11,624. The highest number of reported deaths occurred in 2021 (3809 cases). In the same year, the number of hospitalizations was the highest with a recorded count of 6664 cases. Additionally, in 2022, there were 868 reports of life-threatening outcomes. Furthermore, the category of disability consistently increased each year and reached its highest point in 2021 at a count of 191 reports. Further details on annual outcomes are provided in the Supplementary File.

There were 2729 deaths associated with daratumumab and caused by hematological disorders. This was accompanied by 89 cases of disability, 4477 hospitalizations, and 436 life-threatening situations. Notably, infectious diseases and respiratory disorders led to significant numbers of hospitalizations: 3192 and 3117, respectively. Other categories, such as cardiovascular and neurological disorders, resulted in considerable life-threatening outcomes, namely 254 reports on cardiovascular issues and 316 on neurological problems. Additionally, near misses/medical errors led to 291 deaths (Figure 2 and Supplementary File).

Over time, physicians have been the primary source of reporting AEs related to daratumumab, with a significant surge in reports reaching 17,177 in 2021. Although consumers and healthcare professionals also made notable contributions, particularly in 2022 with 3429 and 4751 reports, respectively, their numbers were considerably smaller compared to those from physicians. It is noteworthy that pharmacists’ involvement in reporting has steadily increased over the years, while lawyers’ contributions remained minimal (Supplementary File).

The reporting of adverse events associated with daratumumab was mainly carried out by physicians, particularly in the areas of ‘Death’, ‘Hospitalization’, and ‘Other Serious’. The counts for these categories were 7587, 15,188, and 27,903, respectively. Consumers and healthcare professionals also actively reported AEs, especially in the categories of ‘Hospitalization’ and ‘Other Serious’. HPs submitted a total of 6343 reports for hospitalizations and 9200 reports for other serious outcomes (Figure 2 and Supplementary File).

### 2.2. Ixazomib

**Figure 3:**
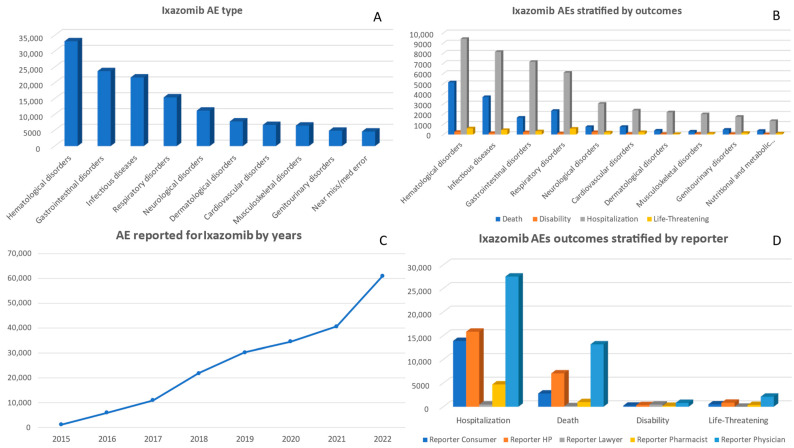
Panel of figures for ixazomib AE type, AEs stratified by outcomes, AEs by years, and outcomes stratified by type of reporter. (**A**) shows the 10 most prevalent AEs that have been reported for ixazomib. (**B**) shows ixazomib’s AEs stratified by its outcomes. (**C**) shows the trend line for ixazomib AEs from 2015 to 2022. (**D**) shows ixazomib’s AE outcomes stratified by type of reporter.

Between 2015 and 2022, a total of 206,243 adverse events associated with ixazomib were reported. The number of reported AEs has consistently increased each year, reaching its highest point in 2022 at 60,835 (Figure 3). Both genders had similar rates of AE reports, with males accounting for 93,786 cases and females for 99,486 cases. Analysis by age group revealed that the majority of AEs occurred in individuals aged between 55–64 (30,069), 65–74 (61,711), and >75 (44,351) age groups (Supplementary File). Between the years of 2015 and 2022, AEs from ixazomib were most commonly associated with the Miscellaneous adverse event category, totaling 51,907 reports (Supplementary File). This was followed by ‘Hematological disorders’, which had a total of 33,171 reports. There were also significant numbers of reported cases of ‘Infectious diseases’ and ‘Gastrointestinal disorders’ with 21,700 and 23,721 reports, respectively (Figure 3).

From 2015 to 2022, there has been a consistent increase in the adverse outcomes associated with ixazomib, as illustrated in Figure 3. The most prevalent reported outcome, referred to as ‘Other Serious’, experienced a significant surge from 414 cases in 2015 to a substantial number of 30,803 cases by 2022 (Supplementary File). Similarly, the occurrence of ‘Hospitalization’ notably rose from 301 cases in the initial year to an alarming total of 17,255 by the end of this period (Figure 3). The overall deaths reported for ixazomib were 24,206 deaths. It is worth noting that an upward trend was observed in the number of deaths linked to ixazomib use; it increased from 345 deaths recorded in 2015 to 9252 in the final year of observation (Supplementary File). Interestingly, while ‘Required Intervention to Prevent Permanent Impairment/Damage’ and ‘Congenital Anomaly’ remained relatively low in frequency, they were inconsistent across the years (Supplementary File). Based on the data presented in Figure 3, it is apparent that ‘Hematological disorders’ exhibited the strongest correlation with mortality, resulting in 5135 cases of death. The second highest association was observed for ‘Infectious diseases’, which led to 3688 deaths. In terms of hospitalization outcomes, ‘Hematological disorders’ (9411 cases) and ‘Gastrointestinal disorders’ (7157 cases) were frequently reported as categories requiring hospitalization.

During the period from 2015 to 2022, physicians submitted the majority of reports on adverse events associated with ixazomib, totaling 82,033. Consumers and health professionals contributed a significant number as well, with 47,920 and 36,459 reports, respectively. In particular, there was an increase in reporting by physicians (23,569) and health professionals (19,827) observed in 2022 (Supplementary File).

Examining Figure 3, the data reveal that physicians were responsible for reporting a significant number of outcomes, particularly ‘Death’ (13,165 cases), ‘Hospitalization’ (27,539 cases), and ‘Other Serious’ (33,048 cases). Consumers predominantly reported ‘Other Serious’ outcomes (23,474 cases) followed by ‘Hospitalization’ (13,885 cases). Strikingly enough, lawyers showed a distinct connection with reporting instances of ‘Disability’ (461 cases) compared to other outcomes.

### 2.3. Elotuzumab

**Figure 4:**
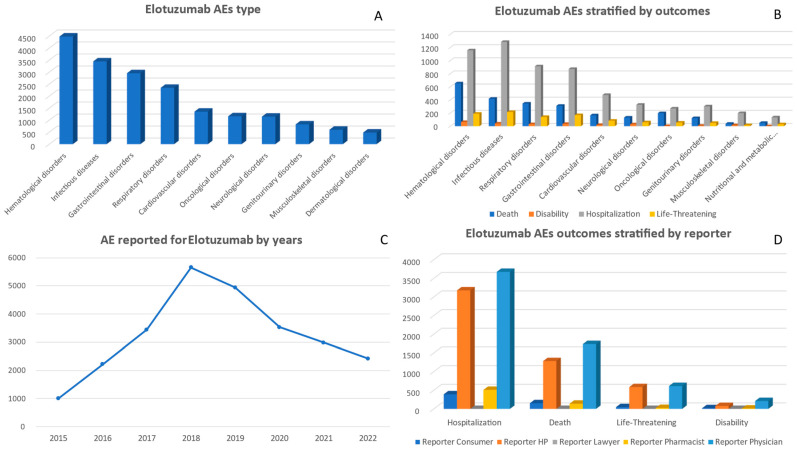
Panel of figures for elotuzumab AE type, AEs stratified by outcomes, AEs by years, and outcomes stratified by type of reporter. (**A**) shows the 10 most prevalent AEs that have been reported for elotuzumab. (**B**) shows elotuzumab’s AEs stratified by its outcomes. (**C**) shows the trend line for elotuzumab AEs from 2015 to 2022. (**D**) shows elotuzumab’s AEs outcomes stratified by type of reporter.

A total of 26,193 adverse events associated with elotuzumab were reported between 2015 and 2022. The highest number of AE reports occurred in 2018, with 5651 cases (Table 1). Subsequently, the number of cases gradually decreased over the following years, reaching 2418 in 2022. Regarding gender distribution among the AE reports, females accounted for 8987 cases, while males accounted for 14,987 cases. When stratifying by age, there was a notable concentration of AE in individuals aged between 65–74 years (7440 cases), followed by those aged between 55 and 64 years (5187 cases) (Supplementary File). Based on the data presented in Figure 4, from 2015 to 2022, the most significant adverse event associated with elotuzumab was categorized as ‘Hematological disorders’, accounting for 4480 reported cases. Following closely behind were reports of ‘Infectious diseases’, with a tally of 3445 reports, and ‘Gastrointestinal disorders’, with approximately 2953 reports. Furthermore, there was notable documentation regarding ‘Cardiovascular disorders’, totaling around 1354 reported cases, and ‘Respiratory disorders’, accounting for approximately 2346 incidences. Conversely, it is important to mention that the category labeled ‘Congenital familial and genetic disorders’ depicted minimal representation across this eight-year timeframe with just a mere total count of only eight recorded cases.

A total of 3349 reported deaths resulted from adverse events associated with elotuzumab between 2015 and 2022. The highest number of deaths occurred in the year 2018 (835 deaths). ‘Other Serious’ outcomes were more prevalent, with a total of 11,528 cases observed. Additionally, there were significant numbers of hospitalizations related to elotuzumab adverse events (7779 cases). On the other hand, instances requiring intervention to prevent permanent impairment or damage were relatively rare and accounted for only three reported cases in 2022. (Figure 4 and Supplementary File).

‘Hematological disorders’ were associated with the highest death report (653), followed by ‘Infectious diseases’ (419) and ‘Cardiovascular disorders’ (166). For the ‘Hospitalization’ outcome, ‘Hematological disorders’ (1156 cases report) and ‘Infectious diseases’ (1284 cases report) were again at the forefront. ‘Life-threatening’ outcomes were frequently reported in the ‘Infectious diseases’ (216 cases) (Figure 4).

Between 2015 and 2022, the largest number of adverse event reports related to elotuzumab were submitted by physicians, totaling 12,530 reports. Other health professionals also contributed significantly, with a total of 5988 reports. Both consumers and health professionals consistently reported adverse events, contributing totals of 1726 and 3415, respectively (Supplementary File).

There was a consistent pattern among physicians in reporting adverse events related to elotuzumab, with the highest number of cases reported for ‘Death’ (1737 cases), ‘Other Serious’ outcomes (5949 cases), and ‘Hospitalization’ (3676 cases). Health professionals also reported a significant number of ‘Hospitalization’ (3177 cases) and ‘Other Serious’ outcomes (3820 cases). Although comparatively lower in number, consumers reported 777 instances of ‘Other Serious’ outcomes. (Figure 4 and Supplementary File).

Upon examining the correlation between specific adverse events category and their reporters, it was observed that physicians had the highest number of reports in several categories. Particularly noteworthy were their reports on ‘Hematological disorders’, with a total of 2866 cases, as well as ‘Infectious diseases’, with 1564 cases. Health professionals also made significant contributions to the reporting of adverse events in the categories of ‘Infectious diseases’ (1512 cases) and ‘Gastrointestinal disorders’ (959 cases). Pharmacists and consumers consistently provided valuable input across multiple categories (Supplementary File).

### 2.4. Panobinostat

Between 2015 and 2022, a cumulative total of 36,448 adverse events associated with panobinostat were reported. There was a significant increase in reports in 2017, with 7072 cases, and in 2020, with 9370 cases (Figure 1). In terms of gender distribution, there were more reports from males compared to females. When looking at different age groups, the majority of AE cases occurred among individuals aged between 55 and 64 years old. (Table 1). Between 2015 and 2022, the highest number of adverse events associated with panobinostat were reported under the category of ‘Hematological disorders’, amounting to a total of 8062 cases. Following that, ‘Gastrointestinal disorders’ had the second-highest count with 5689 reports. Significant counts were also observed for ‘Respiratory disorders’ and ‘Cardiovascular disorders’, which had respective totals of 2092 and 1913 reports (Supplementary File).

**Figure 5:**
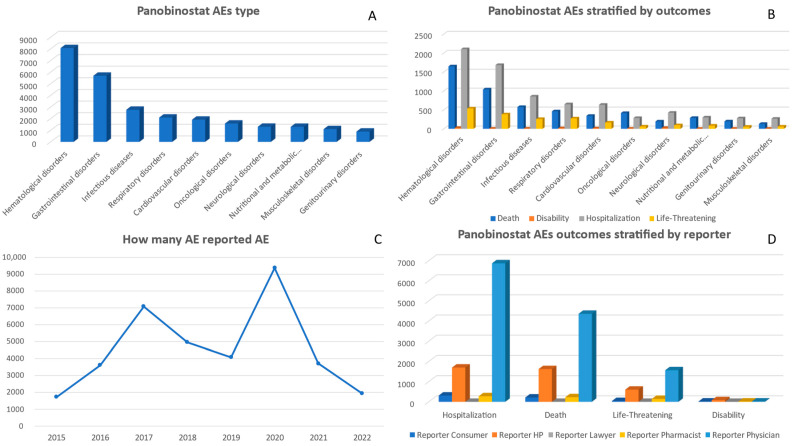
Panel of figures for panobinostat AE type, AEs stratified by outcomes, AEs by years, and outcomes stratified by type of reporter. (**A**) shows the 10 most prevalent AEs that have been reported for panobinostat. (**B**) shows panobinostat’s AEs stratified by its outcomes. (**C**) shows the trend line for panobinostat AEs from 2015 to 2022. (**D**) shows panobinostat’s AEs outcomes stratified by type of reporter.

Analyzing specific adverse event outcomes for panobinostat, the ‘Hematological disorders’ category had the highest number of reported deaths (1650) and was also predominant in ‘Hospitalization’ (2104) and ‘Life-Threatening’ (538) categories. ‘Gastrointestinal disorders’ followed with significant counts across the board, most notably with 1042 deaths and 1684 hospitalizations. Other notable categories include ‘Cardiovascular disorders’ and ‘Infectious diseases’, with substantial reports in death and hospitalization (Figure 5). When examining the adverse event outcomes for panobinostat, it is observed that the category of ‘Hematological disorders’ exhibited the highest number of reported deaths (1650) and was also prominent in terms of hospitalizations (2104) and life-threatening (538) categories. Following closely behind are ‘Gastrointestinal disorders’, which showed significant counts in all categories, including 1042 reported deaths and 1684 hospitalizations. Additionally, there were notable occurrences in the categories of ‘Cardiovascular disorders’ and ‘Infectious diseases’, with many reports indicating death and hospitalization (Figure 5 and Supplementary File).

From 2015 to 2022, panobinostat’s primary source of adverse event reports was physicians, with a total of 22,675 reports. There was a significant rise in physician reports in 2020, reaching a peak at 6912, which marked the highest number among the observed years. Health professionals contributed an additional 7207 reports during this period and experienced their own peak in reporting at 1736 in the year 2020. Consumers and pharmacists provided 1438 and 1033 reports, respectively, during this period (Supplementary File).

In terms of the outcomes of adverse events reported, physicians recorded the highest incidence across all categories. The most severe outcome, ‘Death’, was documented by physicians in 4361 cases, followed by reports from health professionals in 1626 instances. Furthermore, physicians predominantly reported ‘Other Serious’ outcomes, with a total of 9486 reports compared to HP’s 3068 reports. Hospitalizations were also notably higher among physician-reported cases, with a total count reaching 6873 incidents (Figure 5).

When examining specific categories of adverse events, physicians reported a significant number of cases in the category of ‘Hematological disorders’ with 5366 reports. This was closely followed by ‘Gastrointestinal disorders’ with 3619 reports and ‘Cardiovascular disorders’ with 1220 reports, both from physicians. Health professionals also reported high numbers in the ‘Hematological disorders’ category, with 1028 reports, and ‘Infectious diseases’ with 672 reports. It is worth noting that there were no reports in the categories of ‘Congenital, familial and genetic disorders’, as well as ‘Pregnancy, childbirth, and puerperium conditions’ from any reporting group (Supplementary File).

In the disproportionality analysis of multiple myeloma medications, significant associations were observed. Daratumumab was notably associated with respiratory disorders (ROR: 11.865, 95% CI: 11.557–12.180; PRR: 10.929, 95% CI: 10.725–11.137). Ixazomib showed increased reporting of gastrointestinal disorders (ROR: 1.473, 95% CI: 1.441–1.507; PRR: 1.419, 95% CI: 1.407–1.431) and neurological disorders (ROR: 1.146, 95% CI: 1.112–1.180; PRR: 1.138, 95% CI: 1.124–1.152). Elotuzumab was linked with infectious diseases (ROR: 1.363, 95% CI: 1.313–1.415; PRR: 1.316, 95% CI: 1.271–1.361) and cardiovascular disorders (ROR: 1.394, 95% CI: 1.316–1.476; PRR: 1.373, 95% CI: 1.304–1.447). Panobinostat was associated with hematological disorders (ROR: 1.385, 95% CI: 1.349–1.422; PRR: 1.299, 95% CI: 1.269–1.330) and gastrointestinal disorders (ROR: 1.626, 95% CI: 1.577–1.676; PRR: 1.529, 95% CI: 1.489–1.569); see Table 2.

## 3. Discussion

This study highlights the adverse events (AEs) associated with multiple myeloma medications reported in the FAERS database from 2015 to 2022, totaling 367,756 AEs. Ixazomib had the highest number of reports (206,243), followed by daratumumab (98,872), panobinostat (36,448), and elotuzumab (26,193). Most AEs occurred in the 55–74 age group, with unique findings in patients under 18 with 1824 reports for panobinostat in individuals aged 18–24, emphasizing the need for vigilant monitoring and age-specific analysis. This is the first comprehensive analysis of AEs for the full range of new MM medications, providing data stratified by age, year, reporter type, and event type. These insights are crucial for healthcare providers to optimize patient care and for stakeholders to enhance pharmacovigilance practices. The study also evaluates the prevalence and trends of AEs related to novel MM therapies, underscoring the importance of age-specific monitoring and risk mitigation strategies. This analysis lays the foundation for understanding the safety profiles of new MM treatments, enabling personalized patient care and targeted pharmacovigilance efforts.

First, the significant number of adverse events (AEs) reported for multiple myeloma medications underscores the importance of pharmacovigilance and ongoing safety monitoring in clinical practice. Vigilant reporting and analysis are critical for identifying potential safety concerns and enabling prompt intervention. This aligns with the World Health Organization’s emphasis on robust pharmacovigilance systems to safeguard public health and improve drug safety [41]. Given the complex interactions in combination therapies for MM, attentive monitoring is essential, supported by findings from FAERS and additional research studies [42,43]. Postmarketing studies are crucial to detect AEs not observed in early randomized control trials (RCTs). Borrelli and McGladrigan found substantial differences in the safety profiles of MM medications between RCTs and real-world settings [44]. Real-world data often differ from controlled clinical trials, highlighting the significance of databases like FAERS. These databases offer valuable insights into patient responses to medications, including those not captured in formal trials. Our study, along with others such as the one by Mina et al., underscores the crucial role of real-world data in shaping our understanding and informing future therapeutic approaches [45]. Using FAERS, we analyzed a large real-world patient population to quantify the association between different MM medications and AEs.

The finding that ixazomib had the highest number of reported AEs is significant and warrants further investigation. As a proteasome inhibitor used in multiple myeloma treatment, it is crucial to understand the specific AEs associated with ixazomib and identify any high-risk patient populations. Previous research has highlighted the need for tailored monitoring and management of AEs with proteasome inhibitors in multiple myeloma patients [10]. The gender disparity in AE reporting, particularly for daratumumab, is noteworthy and may reflect variations in medication use, response between genders, or differences in reporting behavior. Lee and Wen found that females are underrepresented in cancer clinical trials, impacting the generalizability of safety data [46]. Further research into gender-specific responses to multiple myeloma medications is essential for personalized care. This study reports AEs not only from clinical trials but also from real-world practice and various reporters.

The age-related distribution of AEs also presents important considerations. The higher prevalence of reported AEs in the 55–64 and 65–74 age groups may be attributed to the fact that multiple myeloma is primarily a disease of the elderly [47]. However, the noteworthy number of AEs among patients under 18 in daratumumab and ixazomib and the unusual finding of AEs in the 18–24 age group for panobinostat raise questions about the safety and appropriate use of these medications in younger populations. This highlights the importance of conducting clinical trials and safety assessments specific to pediatric and young adult patients to better understand the risks and benefits of multiple myeloma treatments in these age groups [48].

Daratumumab, a relatively new drug, has proven highly effective for treating multiple myeloma but is associated with an increased risk of AEs, especially in older populations. Since its approval in 2015, the number of reported AEs has significantly increased, peaking in 2021 and slightly decreasing in 2022. This increase is likely due to the drug’s increased use, expanded patient population, and improved AE reporting. The 65–74 age group is the most affected, likely due to the higher prevalence of multiple myeloma, comorbidities, and polypharmacy in this population [49,50,51,52]. The slight decrease in reported AEs in 2022 may result from greater awareness of daratumumab’s potential adverse effects and improved management strategies. Further research is needed to understand the long-term safety of daratumumab and develop strategies to reduce AE risk.

The present study identified reported AEs in MM patients that diverged from the typical utilization trends of MM medications. Alrasheed et al. found that Medicaid claims for daratumumab significantly outnumbered those for ixazomib and elotuzumab. Despite daratumumab’s higher utilization, ixazomib yielded more AE reports, suggesting a higher risk of AEs with ixazomib than with daratumumab in MM patients, even though ixazomib is used less often. One possible explanation is that ixazomib, being an oral medication, is more likely to be taken at home with less supervision, increasing the chance of mistakes. Additionally, oral medications are more prone to interactions with other drugs and foods, contributing to more AEs. Different therapeutic agents for MM have varying risk–benefit profiles. While medications like ixazomib and daratumumab are effective, their benefits must be weighed against possible adverse events. Comparative research, such as studies by Mina et al., highlights the importance of tailoring treatment plans based on individual needs [45].

Although panobinostat was withdrawn from the US market in late 2021, it still has active AE reports from many countries, surpassing AEs of medications like elotuzumab. This suggests panobinostat is still prescribed globally, despite its unconfirmed clinical benefit in the US. The manufacturer could not complete the required post-approval clinical studies to confirm its benefit for MM treatment [33]. This may explain the descending trend of panobinostat AEs in 2021 and 2022. The withdrawal or modification of approved drug uses, as seen with panobinostat and ranitidine, underscores the evolving nature of drug safety information and the importance of post-marketing studies. History shows many medications, like rosiglitazone, rofecoxib, and ranitidine, were withdrawn due to safety concerns, highlighting the need for ongoing surveillance [53,54,55]. Starting in December 2015 allowed for a full year of post-approval data, including a period for market assimilation and AE reporting in the FAERS database. Although panobinostat was approved earlier, our focus was on a comprehensive post-approval view of the latest drugs at the study’s onset. Our study cutoff was December 2022 to account for the time needed for data collection, analysis, and FAERS data processing delays.

Understanding the mechanisms and adverse effects of various multiple myeloma treatments is crucial for optimizing patient care. Proteasome inhibitors like ixazomib disrupt protein degradation, leading to cellular dysfunction and adverse events such as gastrointestinal issues, peripheral neuropathy, thrombocytopenia, and skin reactions. The severity of these effects is influenced by individual patient factors [56,57]. Daratumumab targets CD38 on myeloma and some immune cells, causing immune activation and infusion-related reactions (fever, chills, respiratory issues), as well as hematologic toxicities (neutropenia, thrombocytopenia, anemia), increasing infection risk. Fatigue and other side effects result from complex interactions between CD38 targeting and broader immune effects [58,59]. Elotuzumab targets SLAMF7, causing infusion-related reactions and potentially contributing to cytopenias and infection risk, exacerbated by myeloma-related immunosuppression [12,60]. Panobinostat, a non-selective histone deacetylase inhibitor, disrupts gene expression, leading to gastrointestinal toxicity, bone marrow suppression, potential cardiac arrhythmias, and fatigue from anemia and cellular side effects [61,62].

Further research is needed to compare the safety and efficacy of ixazomib and daratumumab in MM patients. Clinicians should carefully monitor patients on ixazomib for AEs and provide appropriate supportive care. While FAERS database findings offer valuable safety insights, they represent passive surveillance and may have reporting biases. Healthcare professionals and researchers should use these results as a basis for further investigation, including prospective studies and in-depth analyses of specific adverse events. These findings highlight the need for ongoing pharmacovigilance and tailored multiple myeloma treatment strategies for specific patient populations.

## 4. Methods

### 4.1. Study Design

This research adopts a descriptive retrospective approach to comprehensively analyze reports from the FDA Adverse Event Reporting System (FAERS) pertaining to recently approved medications for the treatment of MM.

### 4.2. Data Sources

Data for this study are sourced exclusively from FAERS, covering the expansive timeframe from December 2015 to December 2022. FAERS is a deidentified and publicly accessible dataset, hosted on the US FDA dashboard, that collates valuable information regarding AEs associated with various medications, including those for MM. The FAERS database is a large repository of voluntarily submitted adverse event reports from healthcare professionals, consumers, and pharmaceutical companies. Data quality can vary, and the database is primarily used for identifying potential safety signals rather than proving causation.

### 4.3. Data Components

The dataset harvested from FAERS encompasses an array of pertinent variables, including medication brand names, report date, patient age and gender, AEs with their associated outcomes, and reporter type, like healthcare professional, patient, or lawyer.

### 4.4. Data Curation

The initial dataset is vast, comprising over 3700 distinct AEs. To enhance the clarity and facilitate meaningful comparison, these AEs have been methodically categorized into 21 different groups, aligned with the relevant diseases or conditions they pertain to. A comprehensive list of these categories is furnished in Table 3.

### 4.5. Disproportionality Analysis

A disproportionality analysis was performed to identify potential associations between multiple myeloma medications and the top five AEs using data from FAERS.

Calculation of Measures:

The reporting odds ratio (ROR) and the proportional reporting ratio (PRR) were calculated:

ROR: The odds of reporting an AE for a specific drug were compared with the odds for all other drugs.

*ROR = (A/C)(B/D)*

PRR: The proportion of reports for an AE among all reports for the drug was compared with the proportion for all other drugs.

*PRR = A/(A+C)/B/(B+D)*

where:

A: Number of reports of the specific adverse event for the drug of interest

B: Number of reports of the specific adverse event for all other drugs

C: Number of reports of all other adverse events for the drug of interest

D: Number of reports of all other adverse events for all other drugs

Statistical Significance:

Ninety-five percent confidence intervals were calculated for both ROR and PRR. A signal was considered significant if the lower bound of the confidence interval was greater than 1.

## 5. Strengths and Limitations

This study has several limitations; first, FAERS data rely on voluntary reporting from different resources with significant variation in their background, from consumers to manufacturers to different health care providers, which may introduce underreporting like recall, measurement, and social desirability bias. The quality and completeness of data may vary from one report to another, and not all AEs may be reported. Also, this type of data provide information on the association between a medication and an adverse event, but it does not establish causality. It provides information on reported associations but does not confirm whether the medication directly caused the event. This requires further investigation, such as clinical trials or epidemiological studies. The dataset may not capture all potential confounding factors that could influence the relationship between a medication and an AE. Factors such as underlying health conditions, concomitant medications, and patient demographics may not be adequately controlled for. Moreover, the analysis may not account for temporal trends in AE reporting, changes in reporting practices over time, or changes in regulatory requirements or guidelines. Despite these limitations, this study’s results are derived from real-world reports of adverse events associated with medications. These real-world data can provide insights into the post-market safety and effectiveness of recently approved medications, which may not have been extensively studied in controlled clinical trials. This can help fill gaps in knowledge. The study covers an expansive timeframe and a substantial number of adverse event reports (over 3700 distinct AEs). This large sample size can enhance the statistical power and generalizability of the findings. In line with these limitations, we recommend that clinicians carefully monitor patients of different age groups and consider specific patient demographics when prescribing these medications. Our analysis suggests that particular attention should be given to older adults who may have multiple comorbidities and are often on various medications, which could increase the risk of adverse events. Additionally, clinicians should be vigilant about the potential for underreporting of AEs and proactively inquire about any adverse experiences patients may have. Personalized medicine approaches, including dose adjustments and closer monitoring, may be beneficial for high-risk patient groups.

## 6. Conclusions

This study provides crucial insights into the AE patterns associated with newer MM medications. The exceptionally high number of AEs and deaths reported for ixazomib, along with its significant rise over time, demands urgent attention. This disproportionate safety signal necessitates in-depth mechanistic studies to inform risk mitigation strategies and optimize patient care. While AE distribution was generally even across genders, elotuzumab’s notable male bias warrants further investigation to identify potential sex-specific risk factors. Our findings emphasize the continuous need for pharmacovigilance in multiple myeloma therapies. Moreover, given the focus on older patients in initial drug trials, our results highlight the importance of prioritizing safety monitoring and carefully evaluating these newer agents in younger patient populations, who may experience unique AE profiles. Future clinical trials should include diverse patient demographics, longer follow-up periods, and adaptive designs to capture and address adverse events more effectively. Incorporating real-world evidence and mechanistic studies will help develop targeted risk mitigation strategies and optimize patient care

## Figures and Tables

**Table 1:** AEs reported for daratumumab, ixazomib, elotuzumab, and Panobinostat, stratified by sex and age group.

Medication	Ixazomib (%)	Daratumumab (%)	Panobinostat (%)	Elotuzumab (%)
Total reported AEs	206,243 (56.1)	98,872 (26.9)	36,448 (9.9)	26,193 (7.1)
Female	93,786 (45)	41,279 (42)	11,970 (33)	8987 (34)
Male	99,486 (48)	47,143 (48)	15,132 (41)	14,198 (54)
NA	12,971 (6)	10,450 (11)	9346 (26)	3008 (11)
<18	1478 (0.7)	2043 (2.1)	488 (1.3)	433 (1.7)
18–24	1 (0.0)	62 (0.1)	1824 (5)	4 (0.0)
25–34	146 (0.1)	113 (0.1)	46 (0.1)	18 (0.1)
35–44	1264 (0.6)	1378 (1.4)	1221 (3.3)	159 (0.6)
45–54	5937 (2.9)	5665 (5.7)	789 (2.2)	1620 (6.2)
55–64	30,069 (14.6)	19,975 (20.2)	8786 (24.1)	5187 (19.8)
65–74	61,711 (29.9)	32,032 (32.4)	6345 (17.4)	7440 (28.4)
>75	44,351 (21.5)	13,886 (14)	5418 (14.9)	5280 (20.2)
NA	61,286 (29.7)	23,718 (24)	11,531 (31.6)	6052 (23.1)

**Table 2:** Disproportionality analysis of the top five adverse events associated with multiple myeloma medications.

**Daratumumab**		
**AEs**	**ROR (95% CI)**	**PRR (95% CI)**
Hematological disorders	1.152 (1.130–1.174)	1.123 (1.108–1.138)
Infectious diseases	0.938 (0.915–0.961)	0.944 (0.927–0.961)
Respiratory disorders	11.865 (11.557–12.180)	10.929 (10.725–11.137)
Gastrointestinal disorder	0.571 (0.556–0.586)	0.601 (0.589–0.615)
Neurological disorders	1.053 (1.019–1.088)	1.049 (1.025–1.074)
**Ixazomib**		
**AEs**	**ROR**	**PRR**
Hematological disorders	0.798 (0.785–0.812)	0.831 (0.824–0.837)
Gastrointestinal disorder	1.473 (1.441–1.507)	1.419 (1.407–1.431)
Infectious diseases	1.077 (1.054–1.101)	1.069 (1.059–1.079)
Respiratory disorders	0.926 (0.904–0.949)	0.931 (0.921–0.942)
Neurological disorders	1.146 (1.112–1.180)	1.138 (1.124–1.152)
**Elotuzumab**		
**AEs**	**ROR**	**PRR**
Hematological disorders	0.973 (0.941–1.006)	0.977 (0.948–1.009)
Infectious diseases	1.363 (1.313–1.415)	1.316 (1.271–1.361)
Gastrointestinal disorder	1.059 (1.018–1.103)	1.053 (1.015–1.092)
Respiratory disorders	1.193 (1.141–1.247)	1.176 (1.129–1.224)
Cardiovascular disorders	1.394 (1.316–1.476)	1.373 (1.304–1.447)
**Panobinostat**		
**AEs**	**ROR**	**PRR**
Hematological disorders	1.385 (1.349–1.422)	1.299 (1.269–1.330)
Gastrointestinal disorder	1.626 (1.577–1.676)	1.529 (1.489–1.569)
Infectious diseases	0.698 (0.671–0.727)	0.721 (0.695–0.749)
Respiratory disorders	0.707 (0.675–0.740)	0.724 (0.694–0.755)
Cardiovascular disorders	1.437 (1.367–1.509)	1.414 (1.354–1.476)

**Table 3:** Adverse events categories and its examples as reported from FAERS database.

Adverse Event Category	Examples of AEs Reported
Cardiovascular disorders	Cardiac arrest, Cardiac dysfunction, Chest pain, Palpitations, Aortic aneurysm
Congenital, familial and genetic disorders	Talipes, Wolff–Parkinson–White syndrome, Palatal disorder
Dermatological disorders	Skin plaque, Actinic keratosis, Dermatitis
Endocrine disorders	Adrenal insufficiency, Hypothyroidism Cushing’s syndrome
ENT disorder	Otitis media, Paranasal sinus hypersecretion, Laryngitis
Gastrointestinal disorders	Gastrointestinal obstruction, Fistula of small intestine, Gastric ulcer
Genitourinary disorders	Renal failure, Nephrolithiasis, Urethral pain, Epididymitis
Hematological disorders	Purpura, Hemoglobin decreased, Thrombosis
Immunological disorders	Autoimmune disorder, Type IV hypersensitivity reaction, Antiphospholipid syndrome
Infectious diseases	Enterobacter sepsis, Staphylococcal bacteremia, Escherichia infection
Miscellaneous	Catheter site hemorrhage, Fatigue, Pain, Multiple injuries
Musculoskeletal disorders	Femoral neck fracture, Muscular weakness, Limb discomfort
Near miss/med error	Dose calculation error, Poor quality product administered, Drug interaction
Neurological disorders	Dizziness, Seizure, Cognitive disorder
Nutritional and metabolic disorders	Hyponatremia, Increased appetite, Diet refusal, Anion gap increased
Oncological disorders	Malignant melanoma, Lung cancer, Colon cancer, Lymphoma
Ophthalmic disorders	Retinal disorder, Vision blurred, Cataract
Oral and dental disorders	Mouth ulceration, Gingival swelling, Dental caries
Psychiatric disorders	Depression, Anxiety, Suicidal ideation, Schizophrenia
Respiratory disorders	Bronchitis, Asthma, Pulmonary edema, Bronchospasm

## Data Availability

The dataset supporting the conclusions of this article is available in the FDA Adverse Event Reporting System (FAERS) public dashboard repository, at: https://www.fda.gov/drugs/questions-and-answers-fdas-adverse-event-reporting-system-faers/fda-adverse-event-reporting-system-faers-public-dashboard (accessed on 20 May 2023).

## Supplementary Materials

The following supporting information can be downloaded at: https://www.mdpi.com/article/10.3390/ph17070815/s1, Table S1: Adverse Events (AE) of daratumumab stratified by sex and age from of 2015 until 2022. Table S2: Adverse Events (AE) of daratumumab stratified by the type of AE from 2015 until 2022. Table S3: Adverse Events (AE) of daratumumab stratified by the type of outcomes from 2015 until 2022. Table S4: Adverse Events (AE) of daratumumab stratified by reporter from 2015 until 2022. Table S5: Types of adverse Events (AE) of daratumumab stratified by reporter from 2015 until 2022. Table S6: Types of adverse Events (AE) of daratumumab stratified by the type of outcomes from 2015 until 2022. Table S7: Adverse Events (AE) outcomes of daratumumab stratified by reporter from 2015 until 2022. Table S8: Adverse Events (AE) of ixazomib stratified by sex and age from 2015 until 2022. Table S9: Adverse Events (AE) of ixazomib stratified by the type of AE from 2015 until 2022. Table S10: Adverse Events (AE) of ixazomib stratified by the type of outcomes from 2015 until 2022. Table S11: Adverse Events (AE) of ixazomib stratified by reporter from 2015 until 2022. Table S12: Types of adverse Events (AE) of ixazomib stratified by reporter from 2015 until 2022. Table S13: Types of adverse Events (AE) of ixazomib stratified by the type of outcomes from 2015 until 2022. Table S14: Adverse Events (AE) outcomes of ixazomib stratified reporter from 2015 until 2022. Table S15: Adverse Events (AE) of elotuzumab stratified by sex and age from 2015 until 2022. Table S16: Adverse Events (AE) of elotuzumab stratified by the type of AE from 2015 until 2022. Table S17: Adverse Events (AE) of elotuzumab stratified by the type of outcomes from 2015 until 2022. Table S18: Adverse Events (AE) of elotuzumab stratified by reporter from 2015 until 2022. Table S19: Types of adverse Events (AE) of elotuzumab stratified by reporter from 2015 until 2022. Table S20: Types of adverse Events (AE) of elotuzumab stratified by the type of outcomes from 2015 until 2022. Table S21: Adverse Events (AE) outcomes of elotuzumab stratified by reporter from 2015 until 2022. Table S22: Adverse Events (AE) of panobinostat stratified by sex and age from 2015 until 2022. Table S23: Adverse Events (AE) of panobinostat stratified by the type of AE from 2015 until 2022. Table S24: Adverse Events (AE) of panobinostat stratified by the type of outcomes from 2015 until 2022. Table S25: Adverse Events (AE) of panobinostat stratified by reporter from 2015 until 2022. Table S26: Types of adverse Events (AE) of panobinostat stratified by reporter from 2015 until 2022. Table S27: Types of adverse Events (AE) of panobinostat stratified by the type of outcomes from 2015 until 2022. Table S28: Adverse Events (AE) outcomes of panobinostat stratified by reporter from 2015 until 2022.
