# Supplementary material for: Descriptive Analysis of Adverse Events Reported for New Multiple Myeloma Medications Using FDA Adverse Event Reporting System (FAERS) Databases from 2015 to 2022
Source: Pharmaceuticals (Basel). 2024 Jun 21;17(7):815. doi: 10.3390/ph17070815 (PMC11279559; doi:10.3390/ph17070815)
Supplement: Supplementary file 1 [file pharmaceuticals-17-00815-s001.zip › pharmaceuticals-3025375-supplementary.pdf]

Daratumumab

Reported AEs		Sex		Age Group							
**Year**	AE	Female	Male	< 18	18-24	25-34	35-44	45-54	55-64	65-74	> 75
**2015**	35	7	12	0	0	0	0	7	0	6	0
**2016**	2,871	1,026	1,476	70	0	1	14	176	394	607	357
**2017**	4,917	1,767	2,593	102	0	12	92	328	703	1,017	662
**2018**	12,486	5,983	5,722	186	11	0	55	1,320	2,129	4,795	1,598
**2019**	17,211	7,361	8,601	407	6	15	189	1,252	3,390	6,486	2,008
**2020**	16,620	6,295	8,764	237	1	21	191	786	2,927	5,881	2,450
**2021**	26,542	12,135	11,786	454	2	19	349	804	6,733	9,096	3,678
**2022**	18,190	6,705	8,189	587	42	45	488	992	3,699	4,144	3,133
**Total**	98,872	41,279	47,143	2,043	62	113	1,378	5,665	19,975	32,032	13,886

Table S1: Adverse Events (AE) of daratumumab stratified by sex and age from of 2015 until 2022.

**Year**	**AE Type**																			
	**CVD**	**CF/GD**	**DD**	**ED**	**ENT**	**GIT**	**GUD**	**HD**	**IM**	**ID**	**MSD**	**NM/ME**	**ND**	**NMD**	**OD**	**OPD**	**ODD**	**PD**	**RD**	**MSC**
**2015**	4	0	0	0	0	0	3	0	0	0	0	0	6	1	0	0	0	1	0	20
**2016**	166	0	48	16	20	100	40	326	31	177	108	206	86	31	44	12	5	64	317	1,074
**2017**	273	0	78	60	36	245	84	689	65	325	111	258	256	100	172	39	33	101	435	1,557
**2018**	577	0	220	159	121	904	152	2,358	167	1,000	238	360	546	270	355	92	246	237	1,116	3,368
**2019**	664	1	218	147	128	1,213	221	3,867	110	1,652	498	518	734	308	464	223	177	229	1,726	4,113
**2020**	730	0	365	115	58	1,252	348	3,391	128	1,625	510	476	1,041	233	459	140	172	328	1,246	4,003
**2021**	896	2	541	382	86	2,183	635	5,433	157	2,659	699	698	1,407	511	581	199	402	459	2,196	6,416
**2022**	949	36	450	145	79	1,266	500	2,669	162	2,247	516	537	1,197	239	364	349	106	186	1,479	4,714
**Total**	4,259	39	1,920	1,024	528	7,163	1,983	18,733	820	9,685	2,680	3,053	5,273	1,693	2,439	1,054	1,141	1,605	8,515	25,265

Table S2: Adverse Events (AE) of daratumumab stratifiedby the type of AE from 2015 until 2022.

CVD: Cardiovascular disorders, CF/GD: Congenital, familial and genetic disorders, DD: Dermatological disorders, ED: Endocrine disorders, ENT: Ear nose throat disorder, GIT: Gastrointesti0l disorders, GUD: Genitouri0ry disorders, HD: Hematological disorders, IM: Immunological disorders, ID: Infectious diseases, MSD: Musculoskeletal disorders, NM/ME: Near miss/med error ND: Neurological disorders, NMD: Nutrition and metabolic disorders, OD: Oncological disorders, OPD: Ophthalmic disorders, ODD: Oral and dental disorders, PD: Psychiatric disorders, RD: Respiratory disorders, MSC: Miscellaneous

Year	Outcome						
	Death	Disability	Hospitalization	Life-Threatening	CA	RIPPID	Other Serious
**2015**	2	0	18	6	0	0	9
**2016**	193	12	678	69	0	0	1,122
**2017**	485	79	1,348	246	0	0	1,906
**2018**	1,356	96	3,231	660	0	0	5,835
**2019**	1,512	102	5,099	298	0	0	8,653
**2020**	2,224	150	4,614	489	0	0	7,245
**2021**	3,809	191	6,664	799	12	14	12,269
**2022**	2,043	188	4,455	868	5	23	8,891
**Total**	11,624	818	26,107	3,435	17	37	45,930

Table S3: Adverse Events (AE) of daratumumab stratified by the type of outcomes from 2015 until 2022.
CA: Congenital Anomaly, RIPPID: Required Intervention to Prevent Permeant Impairment/Damage

Year	Reporter				
	**Consumer**	**HP**	**Lawyer**	**Pharmacist**	**Physician**
**2015**	18	6	0	10	1
**2016**	1,137	788	0	447	483
**2017**	1,127	1,735	0	778	1,274
**2018**	1,597	3,293	10	1,412	6,172
**2019**	1,055	3,183	0	1,801	11,131
**2020**	1,052	3,554	0	2,377	9,559
**2021**	1,486	5,045	0	2,803	17,177
**2022**	3,429	4,751	8	1,488	8,442
**Total**	10,901	22,355	18	11,116	54,239

Table S4: Adverse Events (AE) of daratumumab stratified by reporter from 2015 until 2022.
HP: Health professio0l.

AEs	Reporter				
	Consumer	HP	Lawyer	Pharmacist	Physician
**Cardiovascular disorders**	646	975	0	458	2,170
**Congenital, familial and genetic disorders**	0	0	0	3	36
**Dermatological disorders**	289	446	4	408	770
**Endocrine disorder**	97	205	0	108	613
**ENT disorder**	70	196	0	82	180
**Gastrointesti0l disorders**	858	1,727	1	962	3,602
**Genitouri0ry disorders**	229	330	4	157	1,259
**Hematological disorders**	983	2,825	0	1,671	13,190
**Immunological disorders**	160	199	0	127	332
**Infectious diseases**	773	2,447	1	728	5,710
**Musculoskeletal disorders**	384	622	0	327	1,344
**Near miss/med error**	439	911	0	540	1,160
**Neurological disorders**	618	1,731	0	670	2,244
**Nutritio0l and metabolic disorders**	158	321	0	168	1,045
**Oncological disorders**	189	549	2	210	1,484
**Ophthalmic disorders**	173	156	0	135	586
**Oral and dental disorders**	89	91	4	82	873
**Psychiatric disorders**	261	608	0	178	555
**Respiratory disorders**	880	1,705	2	731	5,168
**Miscellaneous**	3,605	8,018	0	3,371	11,918
**Total**	10,901	24,062	18	11,116	54,239

Table S5: Types of adverse Events (AE) of daratumumab stratified by reporter from 2015 until 2022.
HP: Health professio0l

AEs\ Outcomes	Death	Disability	Hospitalization	Life-Threatening
**Cardiovascular disorders**	467	23	1,374	254
**Congenital, familial and genetic disorders**	12	0	0	12
**Dermatological disorders**	76	5	380	26
**Endocrine disorder**	89	2	288	15
**ENT disorder**	25	1	122	11
**Gastrointesti0l disorders**	780	57	1,991	132
**Genitouri0ry disorders**	190	24	774	48
**Hematological disorders**	2,729	89	4,477	436
**Immunological disorders**	43	7	196	60
**Infectious diseases**	1,299	45	3,192	407
**Musculoskeletal disorders**	161	33	845	53
**Near miss/med error**	291	26	526	135
**Neurological disorders**	535	103	1162	316
**Nutritio0l and metabolic disorders**	277	11	490	46
**Oncological disorders**	459	35	412	60
**Ophthalmic disorders**	56	36	217	18
**Oral and dental disorders**	118	4	296	65
**Psychiatric disorders**	185	17	410	61
**Respiratory disorders**	708	38	3117	432
**Miscellaneous**	3,124	262	5,838	848
**Total**	11,624	818	26,107	3,435

Table S6: Types of adverse Events (AE) of daratumumab stratified by the type of outcomes from 2015 until 2022.

Outcomes	Reporter				
	Consumer	HP	Lawyer	Pharmacist	Physician
**Death**	720	2,761	1	508	7,587
**Disability**	90	110	0	96	522
**Hospitalization**	2,200	6,343	6	2,305	15,188
**Life-Threatening**	479	971	0	263	1721
**Other Serious**	4,894	9,200	11	3,838	27,903
**Congenital Anomaly**	0	9	0	0	8
**Req Interv to Prevnt Permnt Impair/Dmg**	5	0	0	32	0
**total**	8,388	19,394	18	7042	52,929

Table S7: Adverse Events (AE) outcomes of daratumumab stratified by reporter from 2015 until 2022.

CA: Congenital Anomaly, RIPPID: Required Intervention to Prevent Permeant Impairment/Damage

HP: Health professio0l

Ixazomib

Reported AEs		Sex		Age Group							
**Year**	**AE**	**Female**	**Male**	**< 18**	**18-24**	**25-34**	**35-44**	**45-54**	**55-64**	**65-74**	**> 75**
**2015**	1,183	339	346	0	0	0	4	79	99	218	267
**2016**	6,018	2,882	2,898	5	0	2	35	206	893	1,290	880
**2017**	11,003	5,136	4,906	6	0	6	108	327	1,163	2,952	2,123
**2018**	21,806	11,076	10,437	63	0	58	239	412	3,908	6,522	6,012
**2019**	30,211	15,534	13,677	387	0	35	195	970	3,800	7,962	8,437
**2020**	34,541	18,093	15,501	225	1	9	86	734	6,411	6,315	8,699
**2021**	40,646	21,054	16,839	488	0	17	195	1,412	6,301	9,888	8,531
**2022**	60,835	19,672	34,882	304	0	19	402	1,797	7,494	26,564	9,402
**Total**	206,243	93,786	99,486	1,478	1	146	1,264	5,937	30,069	61,711	44,351

Table S8: Adverse Events (AE) of ixazomib stratified by sex and age from 2015 until 2022.

Year	AE Type																				
	**CVD**	**CF/GD**	**DD**	**ED**	**ENT**	**GIT**	**GUD**	**HD**	**IM**	**ID**	**MSD**	**NM/ME**	**ND**	**NMD**	**OD**	**OPD**	**ODD**	**PCPC**	**PD**	**RD**	**MSC**
**2015**	127	1	52	38	1	155	50	101	1	103	11	4	85	48	71	2	1	0	39	97	196
**2016**	232	0	249	44	14	914	163	538	38	357	199	207	339	85	87	101	48	0	114	437	1,852
**2017**	437	0	389	132	28	1,460	262	1,355	52	680	376	282	702	229	163	173	62	0	208	782	3,231
**2018**	785	8	892	230	48	3,185	849	3,772	74	1,823	546	346	995	496	420	199	147	4	306	1,677	5,004
**2019**	1,003	14	1,169	341	88	3,673	964	4,784	240	2,519	721	781	1,638	793	676	307	236	4	515	2,333	7,412
**2020**	1,205	9	1,344	289	69	4,267	864	4,667	253	2,996	1,178	1,042	2,072	761	732	421	217	0	459	2,791	8,905
**2021**	1,467	4	1,817	411	145	5,483	829	5,759	226	2,830	1,667	915	2,783	684	820	688	300	6	985	2,083	10,562
**2022**	1,430	6	1,872	318	170	4,584	875	12,195	237	10,392	1,787	1,037	2,600	740	754	736	361	7	778	5,211	14,745
**total**	6,686	42	7,784	1,803	563	23,721	4,856	33,171	1,121	21,700	6,485	4,614	11,214	3,836	3,723	2,627	1,372	21	3,404	15,411	51,907

Table S9: Adverse Events (AE) of ixazomib stratified by the type of AE from 2015 until 2022.

CVD: Cardiovascular disorders, CF/GD: Congenital, familial and genetic disorders, DD: Dermatological disorders, ED: Endocrine disorders, ENT: Ear nose throat disorder, GIT: Gastrointesti0l disorders, GUD: Genitouri0ry disorders, HD: Hematological disorders, IM: Immunological disorders, ID: Infectious diseases, MSD: Musculoskeletal disorders, NM/ME: Near miss/med error ND: Neurological disorders, NMD: Nutritio0l and metabolic disorders, OD: Oncological disorders, OPD: Ophthalmic disorders, ODD: Oral and dental disorders, PCPC: Preg0ncy, childbirth, and puerperium conditions, PD: Psychiatric disorders, RD: Respiratory disorders, MSC: Miscellaneous

Year	outcomes						
	Death	Disability	Hospitalization	Life-Threatening	RIPPID	CA	Other Serious
2015	345	0	301	76	0	0	414
2016	430	95	1,613	111	1	5	1,730
2017	1,239	136	3,302	151	1	0	3,701
2018	2,409	209	7,974	581	0	0	7,734
2019	3,051	474	10,367	614	5	1	11,526
2020	4,259	479	10,637	1,170	4	2	12,797
2021	3,221	393	11,046	362	0	1	20,552
2022	9,252	253	17,255	789	0	2	30,803
total	24,206	2,039	62,495	3,854	11	11	89,257

Table S10: Adverse Events (AE) of ixazomib stratified by the type of outcomes from 2015 until 2022.
CA: Congenital Anomaly, RIPPID: Required Intervention to Prevent Permeant Impairment/Damage

Year	Reporter				
	Consumer	HP	Lawyer	Pharmacist	Physician
2015	24	724	0	34	397
2016	1,157	2,246	0	385	2,159
2017	3,241	3,554	8	895	3,302
2018	4,172	4,138	1	2,557	10,919
2019	5,746	7,137	595	3,383	13,265
2020	7,085	6,581	923	3,829	16,023
2021	13,157	10,032	0	4,803	12,399
2022	13,338	19,827	2	4,079	23,569
Total	47,920	54,239	1,529	19,965	82,033

Table S11: Adverse Events (AE) of ixazomib stratified by reporter from 2015 until 2022.
HP: Health Professio0l

AEs	Reporter				
	Consumer	HP	Lawyer	Pharmacist	Physician
**Cardiovascular disorders**	1,487	1,771	14	767	2,628
**Congenital, familial and genetic disorders**	15	4	0	1	22
**Dermatological disorders**	1,871	1,907	132	1,033	2,831
**Endocrine disorder**	462	373	34	178	755
**ENT disorder**	191	154	0	99	119
**Gastrointesti0l disorders**	6,036	5,791	118	2,725	8,382
**Genitouri0ry disorders**	1,049	1,074	26	412	2,291
**Hematological disorders**	5,368	8,464	172	2,115	17,028
**Immunological disorders**	254	286	20	120	441
**Infectious diseases**	3,490	6,484	160	1,273	10,273
**Musculoskeletal disorders**	1,955	1,876	29	800	1,807
**Near miss/med error**	1,739	977	0	638	1,254
**Neurological disorders**	3,010	3,115	243	1,307	3,523
**Nutritio0l and metabolic disorders**	740	884	40	251	1,920
**Oncological disorders**	534	1,191	26	328	1,618
**Ophthalmic disorders**	796	735	26	395	672
**Oral and dental disorders**	353	296	46	192	484
**Preg0ncy, childbirth, and puerperium conditions**	4	3	0	2	12
**Psychiatric disorders**	1,058	1,047	26	413	856
**Respiratory disorders**	2,706	4,120	56	1,221	7,283
**Miscellaneous**	14,803	13,687	361	5,695	17,234
**Total**	47,921	54,239	1,529	19,965	81,433

Table S12: Types of adverse Events (AE) of ixazomib stratified by reporter from 2015 until 2022.
HP: Health Professio0l

AEs\ Outcomes	Death	Disability	Hospitalization	Life-Threatening
**Cardiovascular disorders**	760	75	2,378	233
**Congenital, familial and genetic disorders**	2	5	9	1
**Dermatological disorders**	393	71	2,188	50
**Endocrine disorder**	181	23	536	37
**ENT disorder**	14	18	117	8
**Gastrointesti0l disorders**	1,661	229	7,157	319
**Genitouri0ry disorders**	477	65	1,747	165
**Hematological disorders**	5,135	268	9,411	609
**Immunological disorders**	71	11	329	14
**Infectious diseases**	3,688	148	8,134	447
**Musculoskeletal disorders**	299	76	2,006	86
**Near miss/med error**	216	32	951	16
**Neurological disorders**	754	238	3,038	209
**Nutritio0l and metabolic disorders**	370	41	1,335	107
**Oncological disorders**	552	30	758	59
**Ophthalmic disorders**	70	63	552	18
**Oral and dental disorders**	61	23	360	25
**Preg0ncy, childbirth, and puerperium conditions**	1	1	4	-
**Psychiatric disorders**	213	43	867	19
**Respiratory disorders**	2,335	123	6,088	575
**Miscellaneous**	6,953	456	14,530	857

Table S13: Types of adverse Events (AE) of ixazomib stratified by the type of outcomes from 2015 until 2022.

Outcomes	Reporter				
	Consumer	HP	Lawyer	Pharmacist	Physician
Death	2,799	7,056	134	984	13,165
Disability	235	347	461	182	814
Hospitalization	13,885	15,835	462	4,721	27,539
Life-Threatening	501	845	5	372	2,131
Other Serious	23,474	23,794	466	8,346	33,048
Congenital Anomaly	0	6	0	0	5
Req Interv to Prevnt Permnt Impair/Dmg	0	4	0	7	0
total	40,894	47,887	1,528	14,612	76,702

Table S14: Adverse Events (AE) outcomes of ixazomib stratified reporter from 2015 until 2022.

CA: Congenital Anomaly, RIPPID: Required Intervention to Prevent Permeant Impairment/Damage

HP: Health Professio0l

Elotuzumab

Reported AEs		Sex		Age Group							
**YEAR**	AE	Female	Male	< 18	18-24	25-34	35-44	45-54	55-64	65-74	> 75
**2015**	1,003	262	366	0	0	0	0	22	102	291	200
**2016**	2,222	811	875	7	0	0	2	107	300	450	366
**2017**	3,445	1,420	1,533	20	0	0	26	230	529	947	746
**2018**	5,651	1,621	3,868	32	4	8	15	477	1,312	2,088	1,107
**2019**	4,941	1,785	2,673	41	0	6	17	295	911	1,492	1,118
**2020**	3,527	1,393	1,936	88	0	1	56	161	637	952	761
**2021**	2,986	918	1,726	170	0	2	25	88	809	634	599
**2022**	2,418	777	1,221	75	0	1	18	240	587	586	383
**total**	26,193	8,987	14,198	433	4	18	159	1,620	5,187	7,440	5,280

Table S15: Adverse Events (AE) of elotuzumab stratified by sex and age from 2015 until 2022.

Year	AE type																				
	CVD	CF/GD	DD	ED	ENT	GIT	GUD	HD	IM	ID	MSD	NM/ME	ND	NMD	OD	OPD	ODD	PCPC	PD	RD	MSC
2015	47	0	28	5	6	80	39	141	8	119	20	1	38	37	74	6	0	0	20	124	210
2016	133	0	56	27	6	256	98	245	8	246	50	31	63	51	123	25	15	0	53	221	515
2017	187	0	82	45	8	325	81	675	16	326	62	29	106	81	171	17	13	1	36	305	879
2018	261	3	75	91	31	865	229	1,057	23	754	151	46	210	92	163	28	19	0	30	490	1,033
2019	257	2	95	53	3	686	115	802	27	575	106	68	195	85	271	59	29	0	64	428	1,021
2020	170	2	55	48	10	317	120	625	45	527	106	26	195	39	144	28	16	0	41	292	721
2021	190	1	54	46	17	251	103	474	21	447	66	35	174	50	85	40	15	0	29	295	593
2022	109	0	37	17	3	173	39	461	41	451	40	38	165	27	129	40	5	0	10	191	442
Total	1,354	8	482	332	84	2,953	824	4,480	189	3,445	601	274	1,146	462	1,160	243	112	1	283	2,346	5,414

Table S16: Adverse Events (AE) of elotuzumab stratified by the type of AE from 2015 until 2022.

CVD: Cardiovascular disorders, CF/GD: Congenital, familial and genetic disorders, DD: Dermatological disorders, ED: Endocrine disorders, ENT: Ear nose throat disorder, GIT: Gastrointesti0l disorders, GUD: Genitouri0ry disorders, HD: Hematological disorders, IM: Immunological disorders, ID: Infectious diseases, MSD: Musculoskeletal disorders, NM/ME: Near miss/med error ND: Neurological disorders, NMD: Nutritio0l and metabolic disorders, OD: Oncological disorders, OPD: Ophthalmic disorders, ODD: Oral and dental disorders, PD: Preg0ncy, childbirth, and puerperium conditions, PD: Psychiatric disorders, RD: Respiratory disorders, MSC: Miscellaneous

Year	outcomes					
	Death	Disability	Hospitalization	Life-Threatening	Other Serious	Req Interv to Prevnt Permnt Impair/Dmg
2015	164	16	304	47	458	0
2016	294	21	637	99	934	0
2017	572	55	937	136	1,511	0
2018	835	33	1,814	326	2,404	0
2019	570	48	1,434	200	2,251	0
2020	343	77	1,173	193	1,401	0
2021	320	57	943	119	1,261	0
2022	251	14	537	155	1,308	3
Total	3,349	321	7,779	1,275	11,528	3

Table S17: Adverse Events (AE) of elotuzumab stratified by the type of outcomes from 2015 until 2022.

CA: Congenital Anomaly, RIPPID: Required Intervention to Prevent Permeant Impairment/Damage

Year	Reporter				
	Consumer	HP	Lawyer	Pharmacist	Physician
**2015**	24	346	0	0	310
**2016**	431	1,201	0	103	487
**2017**	403	1,092	1	218	1,729
**2018**	166	1,818	0	419	3,236
**2019**	224	1,531	0	445	2,731
**2020**	164	1,067	0	477	1,818
**2021**	76	1,331	0	370	1,203
**2022**	238	1,017	0	146	1,016
**Total**	1,726	9,403	1	2,178	12,530

Table S18: Adverse Events (AE) of elotuzumab stratified by reporter from 2015 until 2022.

HP: Health Professio0l

AEs	Reporter				
	Consumer	HP	Lawyer	Pharmacist	Physician
**Cardiovascular disorders**	87	602	0	110	536
**Congenital, familial and genetic disorders**	1	5	0	0	2
**Dermatological disorders**	23	169	0	65	207
**Endocrine disorder**	18	160	0	26	125
**ENT disorder**	6	34	0	12	26
**Gastrointesti0l disorders**	208	959	1	214	1,527
**Genitouri0ry disorders**	32	297	0	37	456
**Hematological disorders**	204	1,097	0	263	2,866
**Immunological disorders**	10	70	0	32	71
**Infectious diseases**	112	1512	0	228	1564
**Musculoskeletal disorders**	69	138	0	108	275
**Near miss/med error**	32	109	0	78	55
**Neurological disorders**	98	448	0	129	459
**Nutritio0l and metabolic disorders**	23	203	0	33	188
**Oncological disorders**	56	393	0	38	655
**Ophthalmic disorders**	25	94	0	29	92
**Oral and dental disorders**	8	44	0	27	33
**Preg0ncy, childbirth, and puerperium conditions**	0	0	0	1	0
**Psychiatric disorders**	57	100	0	37	75
**Respiratory disorders**	101	967	0	117	1,133
**Miscellaneous**	556	2,002	0	596	2,185
**Total**	1,726	9,403	1	2,180	12,530

Table S19: Types of adverse Events (AE) of elotuzumab stratified by reporter from 2015 until 2022.
HP: Health Professio0l

AEs\ Outcomes	Death	Disability	Hospitalization	Life-Threatening
**Cardiovascular disorders**	166	19	476	84
**Congenital, familial and genetic disorders**	1	2	1	0
**Dermatological disorders**	15	3	120	6
**Endocrine disorder**	55	0	79	17
**ENT disorder**	2	4	24	2
**Gastrointesti0l disorders**	311	35	872	169
**Genitouri0ry disorders**	124	7	303	50
**Hematological disorders**	653	64	1,156	191
**Immunological disorders**	6	0	69	16
**Infectious diseases**	419	41	1,284	216
**Musculoskeletal disorders**	37	15	200	15
**Near miss/med error**	7	0	34	3
**Neurological disorders**	132	29	327	60
**Nutritio0l and metabolic disorders**	51	0	135	30
**Oncological disorders**	199	3	270	55
**Ophthalmic disorders**	9	3	70	1
**Oral and dental disorders**	3	2	22	0
**Psychiatric disorders**	22	6	55	9
**Respiratory disorders**	345	31	912	141
**Miscellaneous**	792	57	1,370	210

Table S20: Types of adverse Events (AE) of elotuzumab stratified by the type of outcomes from 2015 until 2022.

Outcomes	Reporter				
	Consumer	HP	Lawyer	Pharmacist	Physician
**Death**	153	1,283	0	139	1737
**Disability**	19	79	0	10	213
**Hospitalization**	392	3,177	0	511	3,676
**Life-Threatening**	48	586	0	26	615
**Other Serious**	777	3,820	1	688	5,949
**Congenital Anomaly**	0	0	0	0	0
**Req Interv to Prevnt Permnt Impair/Dmg**	0	0	0	3	0
**Total**	1,389	8,945	1	1,377	12,190

Table S21: Adverse Events (AE) outcomes of elotuzumab stratified by reporter from 2015 until 2022.

HP: Health Professio0l

CA: Congenital Anomaly, RIPPID: Required Intervention to Prevent Permeant Impairment/Damage

Panobinostat

Reported AEs		Sex		Age Group							
Year	AE	Female	Male	< 18	18-24	25-34	35-44	45-54	55-64	65-74	> 75
**2015**	1,725	634	831	18	4	30	9	49	411	382	190
**2016**	3,604	1,145	1,907	42	0	3	93	196	459	1,134	712
**2017**	7,072	2,184	4,387	21	8	3	34	146	1,929	1,974	2,082
**2018**	4,955	2,156	2,619	296	0	4	120	148	1,840	1,100	931
**2019**	4,085	1,556	1,163	24	28	0	15	131	474	1,263	425
**2020**	9,370	2,585	2,419	35	1,545	5	39	18	2,282	250	800
**2021**	3,706	551	1,324	13	239	0	3	68	1,086	164	276
**2022**	1,931	1,159	482	39	0	1	908	33	305	78	2
**total**	36,448	11,970	15,132	488	1,824	46	1,221	789	8,786	6,345	5,418

Table S22: Adverse Events (AE) of panobinostat stratified by sex and age from 2015 until 2022.

Year	AE Type																			
	CVD	CF/GD	DD	ED	ENT	GIT	GUD	HD	IM	ID	MSD	NM/ME	ND	NMD	OD	OPD	ODD	PCPC	PD	RD	MSC
**2015**	116	0	16	21	3	343	33	293	11	109	31	6	102	37	40	1	14	0	23	103	423
**2016**	247	0	27	25	2	579	54	917	14	268	51	23	139	154	113	3	8	0	25	257	698
**2017**	330	0	69	132	4	1,251	217	1,674	21	366	176	61	307	360	271	12	53	0	71	486	1,211
**2018**	275	0	9	98	14	765	131	1,270	29	334	125	11	215	231	189	30	9	0	31	259	930
**2019**	168	0	16	54	15	639	98	1,045	1	318	43	94	138	152	190	25	7	0	18	342	722
**2020**	445	0	135	206	10	1,459	262	2,108	34	878	206	80	280	215	408	68	0	0	67	516	1,993
**2021**	320	0	30	211	16	584	83	548	37	377	105	50	73	134	172	8	8	0	1	116	833
**2022**	12	0	17	10	0	69	6	207	0	114	357	7	45	11	194	5	0	0	187	13	677
**Total**	1,913	0	319	757	64	5,689	884	8,062	147	2,764	1,094	332	1,299	1,294	1,577	152	99	0	423	2,092	7,487

Table S23: Adverse Events (AE) of panobinostat stratified by the type of AE from 2015 until 2022.

CVD: Cardiovascular disorders, CF/GD: Congenital, familial and genetic disorders, DD: Dermatological disorders, ED: Endocrine disorders, ENT: Ear nose throat disorder, GIT: Gastrointesti0l disorders, GUD: Genitouri0ry disorders, HD: Hematological disorders, IM: Immunological disorders, ID: Infectious diseases, MSD: Musculoskeletal disorders, NM/ME: Near miss/med error ND: Neurological disorders, NMD: Nutritio0l and metabolic disorders, OD: Oncological disorders, OPD: Ophthalmic disorders, ODD: Oral and dental disorders, PD: Preg0ncy, childbirth, and puerperium conditions, PD: Psychiatric disorders, RD: Respiratory disorders, MSC: Miscellaneous

Year	Outcomes				
	Death	Disability	Hospitalization	Life-Threatening	Other Serious
**2015**	223	29	575	61	551
**2016**	756	33	788	218	1,617
**2017**	1,582	37	1,903	506	2,949
**2018**	918	12	1,544	245	2,177
**2019**	991	15	980	458	1,583
**2020**	1,766	13	3,125	586	3,827
**2021**	766	0	1,003	450	1,477
**2022**	225	0	253	79	1,272
**Total**	7,227	139	10,171	2,603	15,453

Table S24: Adverse Events (AE) of panobinostat stratified by the type of outcomes from 2015 until 2022.

Year	Reporter			
	Consumer	HP	Pharmacist	Physician
**2015**	428	540	46	700
**2016**	221	655	73	2,226
**2017**	190	976	103	4,711
**2018**	136	488	136	2,828
**2019**	69	1,197	66	1,710
**2020**	163	1,736	424	6,912
**2021**	113	942	179	2,455
**2022**	118	673	6	1,133
**Total**	1,438	7,207	1,033	22,675

Table S25: Adverse Events (AE) of panobinostat stratified by reporter from 2015 until 2022.
HP: Health Professio0l

AEs	Reporter				
	Consumer	HP	Lawyer	Pharmacist	Physician
**Cardiovascular disorders**	64	454	0	72	1,220
**Congenital, familial and genetic disorders**	0	0	0	0	0
**Dermatological disorders**	17	67	0	0	197
**Endocrine disorder**	49	152	0	6	456
**ENT disorder**	4	12	0	2	46
**Gastrointesti0l disorders**	273	904	0	92	3,619
**Genitouri0ry disorders**	14	111	0	56	620
**Hematological disorders**	208	1,028	0	138	5,366
**Immunological disorders**	4	32	0	3	108
**Infectious diseases**	49	672	0	114	1761
**Musculoskeletal disorders**	74	210	0	36	691
**Near miss/med error**	29	166	0	2	98
**Neurological disorders**	68	252	0	46	841
**Nutritio0l and metabolic disorders**	19	173	0	12	948
**Oncological disorders**	46	492	0	23	813
**Ophthalmic disorders**	5	36	0	2	107
**Oral and dental disorders**	3	23	0	1	56
**Preg0ncy, childbirth, and puerperium conditions**	0	0	0	0	0
**Psychiatric disorders**	30	136	0	4	241
**Respiratory disorders**	48	568	0	137	1,165
**Miscellaneous**	434	1,719	0	287	4,322
**Total**	1,438	7,207	0	1,033	22,675

Table S26: Types of adverse Events (AE) of panobinostat stratified by reporter from 2015 until 2022.
HP: Health Professio0l

AEs\ Outcomes	Death	Disability	Hospitalization	Life-Threatening
**Cardiovascular disorders**	343	13	635	166
**Congenital, familial and genetic disorders**	0	0	0	0
**Dermatological disorders**	21	4	125	8
**Endocrine disorder**	121	0	285	40
**ENT disorder**	8	0	20	4
**Gastrointesti0l disorders**	1,042	5	1,684	383
**Genitouri0ry disorders**	197	4	279	56
**Hematological disorders**	1,650	23	2,104	538
**Immunological disorders**	23	3	48	19
**Infectious diseases**	576	7	854	259
**Musculoskeletal disorders**	130	4	267	60
**Near miss/med error**	97	0	39	2
**Neurological disorders**	194	23	425	96
**Nutritio0l and metabolic disorders**	288	2	299	85
**Oncological disorders**	417	6	285	62
**Ophthalmic disorders**	18	0	36	12
**Oral and dental disorders**	22	2	28	8
**Preg0ncy, childbirth, and puerperium conditions**	0	0	0	0
**Psychiatric disorders**	47	0	81	16
**Respiratory disorders**	463	15	649	275
**Miscellaneous**	1,570	28	2,028	514

Table S27: Types of adverse Events (AE) of panobinostat stratified by the type of outcomes from 2015 until 2022.

Outcomes	Reporter				
	Consumer	HP	Lawyer	Pharmacist	Physician
**Death**	216	1,626	0	228	4,361
**Disability**	17	84	0	12	17
**Hospitalization**	314	1,701	0	276	6,873
**Life-Threatening**	41	604	0	144	1,569
**Other Serious**	564	3,068	0	316	9,486
**Congenital Anomaly**	0	0	0	0	0
**Req Interv to Prevnt Permnt Impair/Dmg**	0	0	0	0	0

Table S28: Adverse Events (AE) outcomes of panobinostat stratified by reporter from 2015 until 2022.

HP: Health Professio0l
